# Contextual considerations in implementing problem-based learning approaches in a Brazilian medical curriculum: the UNAERP experience

**DOI:** 10.3402/meo.v19.24366

**Published:** 2014-06-13

**Authors:** Reinaldo Bulgarelli Bestetti, Lucélio Bernardes Couto, Gustavo Salata Romão, Guilherme Teixeira Araújo, Carolina Baraldi A. Restini

**Affiliations:** UNAERP Medicine School, University of Ribeirão Preto, Ribeirão Preto, Brazil

**Keywords:** problem-based learning, peer tutoring, integrated curriculum, curriculum development, tutorial session, medical skills, primary care

## Abstract

**Background:**

Despite being a well-established pedagogical approach in medical education, the implementation of problem-based learning (PBL) approaches hinges not only on educational aspects of the medical curriculum but also on the characteristics and necessities of the health system and the medical labor market within which it is situated.

**Aim:**

To report our experiences implementing a PBL-based approach in a region of Brazil where: 1) all pre-university education and the vast majority of medical courses are based on traditional, lecture-based instructions; and 2) students’ career interests in primary care, arguably the prototypical PBL trainee, are heavily disfavored because of economics.

**Results:**

Brazilian guidelines require that clinical training take place during the last 2 years of the medical program and include intensive, supervised, inpatient and outpatient rotations in pediatrics, family medicine, obstetrics and gynecology, internal medicine, and surgery. Throughout the pre-clinical curriculum, then, students learn to deal with progressively more difficult and complex cases – typically through the use of PBL tutors in a primary care context. However, because of curricular time constraints in the clerkships, and students’ general preoccupation with specialty practice, the continuation of PBL-based approaches in the pre-clinical years – and the expansion of PBL into the clerkships – has become exceedingly difficult.

**Discussion and conclusion:**

Our experience illustrates the importance of context (both cultural and structural) in implementing certain pedagogies within one Brazilian training program. We plan to address these barriers by: 1) integrating units, whenever possible, within a spiral curriculum; 2) introducing real patients earlier in students’ pre-clinical coursework (primarily in a primary care setting); and 3) using subject experts as PBL tutors to better motivate students.

Introduced at McMaster University in the late sixties for students already trained in higher education (1), problem-based learning (PBL) has become a well-established approach in medical education. Underlying PBL are principles of construction, self-direction, collaboration, and contextual study (2).

In brief, the process of knowledge construction is based on activation of prior knowledge and integration of new information (elaboration) by group discussion of a problem under the guidance of a tutor – similar to what might be seen among medical teams in clinical practice. Typically, the tutor is a member of the professorial staff who is specially trained to facilitate the learning process (i.e., guiding learners on how to get the information needed). Thus, the PBL approach is student-centered, not teacher-centered (3, 4).

During the tutorial session, after discussing the subject to activate previous knowledge, students establish learning objectives for self-study by assessing what is known and not known relative to the problem (2). The similarity of the problem with a real clinical scenario is thought to facilitate the transfer of knowledge (contextualized study). Following self-guided study, the final, ‘reporting’ phase of the tutorial session brings students together to solve the problem – maximizing the learning of all group members (collaborative study). This, in turn, promotes maximum knowledge retention (3).

On the basis of the above outline, PBL is believed to better prepare medical students for: 1) working in a health care team; 2) developing better doctor–patient relationships; and 3) enhancing clinical problem-solving skills. In addition, PBL is foundational for lifelong learning (5–9).

Although many outcomes of graduates trained in a PBL framework are similar to those produced from traditional lecture-based learning (LBL) formats (10, 11), PBL is widely used in medical education throughout the world. Nevertheless, the implementation of such approaches is not easy and is affected not only by ‘educational’ aspects but also by contextual forces such as the prevailing health system and medical labor market.

In this paper, we report our experience implementing PBL-based methods in our medical training program, emphasizing adaptations we made to counter the dominant presence of lecture-based approaches in all regional pre-university instruction. Finally, we also discuss our PBL-related experiences in the context of Brazilian national guidelines governing medical courses.

## National guidelines for Brazilian medical curricula

In Brazil, about 75% of available medical training is provided by private educational institutions. Similar to the Liaison Committee on Medical Education in North America, Brazilian programs must comply with the 2001 National Guidelines for Medical Courses of the Ministry of Education – meaning that the vast majority of medical programs, including ours, must abide by formally specified guidelines in constructing any medical curricula.

These guidelines require that the focus of the learning process include health promotion, prevention, and treatment of the most prevalent diseases in the country – as well as on health recovery. Moreover, the guidelines require that about 35% of the total workload of medical curricula in private universities is dedicated to clerkship during the last 2 years – when students must train in pediatrics, public health, obstetrics and gynecology, internal medicine, and surgery rotations. Furthermore, the guidelines strongly encourage the use of active learning approaches (like PBL) in medical teaching (12). The national guidelines also mandate students’ exposure to patients of differing levels of clinical complexity, difficulty, and assistance (primary, secondary, and tertiary) – with emphasis on primary and secondary levels. The guidelines strongly suggest that students see patients as early as possible during the medical program.

To monitor compliance with these guidelines, the Ministry of Education evaluates the quality of private medical programs every 3 years – relying heavily on graduates’ performance on a written national examination. Based on this, programs receive a score from 1 to 5, with scores of 2 or below resulting in mandatory programmatic changes as determined by the Ministry of Education. In the case of consistently poor performance, medical training programs may ultimately be closed.

## The Brazilian Health System

The Brazilian Public Health System (BPHS), established by the 1988 Brazilian Constitution, is responsible for the vast majority of health care, serving about 70% of Brazilians (13, 14). The remaining 30% of persons are cared for by alternative private services. Legally, the BPHS is committed to principles of universality of access, integrality of assistance, equity of resources, and community participation in decision-making processes (15). Knowledge of these key BPHS principles is required of all medical programs, and they are typically presented to the students early on in their medical training.

Within the BPHS, the Family Health Program (FHP) reflects the most prominent model of primary care. Covering an estimated 100 million Brazilians (16), the FHP centers rely on health care teams composed of a family physician, a nurse, and 6–10 community health workers who are responsible for periodically visiting ~2,000 families (or~5,000 resident) living within a limited area or district (16).

As a result of its expanding influence, the FHP requires that health professionals be prepared to: 1) care for both families and communities; 2) work in health care teams (17); and 3) focus on primary care, health and epidemiologic indicators, and BPHS principles and policies (13). For these reasons, the general practitioner is a priority in Brazil.

Contrary to this public health and primary care emphasis, however, newly graduated physicians prefer to work in private services, which provide the most lucrative economic opportunities. This obvious disconnect causes graduates to seriously reconsider embarking on careers in primary care and accounts for the growing popularity of medical specialization. Because state regulations do not extend to specialty choice, less than 1% of practicing physicians have been trained in Family Medicine (18).

## The learning process in Brazil

According to the Program for International Student Assessment, which evaluates the high school performance of students around the world, the 2013 results showed Brazil ranked 58th among all countries – down from 53rd place in the previous examination (19). Unfortunately, this suggests marked deficiencies in Brazilian elementary and high school education and, quite potentially, in higher education. As stated, the dominant pedagogy in elementary and middle school continues to be LBL – which emphasizes stimulating teaching tools, but little stimulus to learn.

In general, Brazilian students experience abrupt changes going from elementary and middle school to the academic universe. This becomes more complicated with a marked change in the general approach to learning, such as from LBL to PBL (constructivist, self-directed, collaborative, and contextualized study). Despite the fact that an increasing number of Brazilian universities feature active learning approaches, there remains a clear predominance of more traditional methods (20) – suggesting that students’ transitions from one overarching pedagogical framework to another will continue to be rocky.

Because Brazilian students, on average, enter medical school at age 17, many lack the confidence and maturity needed to excel in PBL-based course work. Especially early on, this adds to the general ‘shock’ of adjusting to professional school, and may further affect students’ motivation to study, their capacity for integrative and contextualized learning, and their understanding of the general instructional approach. Not surprisingly, it is common for Brazilian medical students to be anxious and insecure about their short-term learning in the context of a PBL-based teaching approach.

## The Ribeirão Preto University medical curriculum

Across all eight stages, the medical curriculum consists of tutoring, medical skills, and primary care units – which are linked together wherever possible to better accommodate an integrated, PBL method.

### Tutoring unit

This unit comprises three modules per semester; each module is composed of six problems (on average) related to most prevalent diseases. So, at the end of the eighth stage, immediately before the beginning of clerkship training, each student will participate in 24 modules and solve about 124 problems.

As mentioned, the shift from a traditional teacher-directed instructional paradigm to a student-centered learning paradigm requires some adaptation for students accustomed to more structured, passive learning processes (21). Changes in learning, maturity, and organization are often needed to offset the false impression by some that complex topics have not been completely covered in sufficient detail (20). To minimize these difficulties, a coordinated mix of lectures, simulations of tutorial sessions, and visits to laboratories and clinics are used during the first week of class to introduce the student to this new context.

For example, lectures highlight the historical aspects of PBL and how the medical curriculum will progress from beginning to end – summarizing the three pre-clinical units (tutoring, medical skills, and primary care) as well as the various clerkship rotations (e.g., pediatrics, obstetrics and gynecology, surgery, internal medicine, and primary care). The laboratory visits, particularly related to medical skills, are intended to show students where the practice of a specific subject discussed in the tutorial session will take place.

Another difficulty for Brazilian students adapting to the PBL method is the motivational aspect of preparing for the tutorial session. Because PBL emphasizes a reflexive, self-directed, and collaborative learning process (22) that requires both student autonomy and responsibility (23), failing to study the learning goals set forth in the tutorial session can interfere with the overall group learning. In this respect, an unprepared student can negatively interfere with the group by compromising the process of mutual dependency during the reporting phase (24). To avoid this, and to improve students’ self-guided study, we administer multiple-choice tests before and after the reporting phase of the tutorial session (25).

Also worth noting is that the tutor must scaffold the learning process (26) by guiding students to learn; that is, motivating them to construct their knowledge and discover how to research and use these means (20). These requirements are unfamiliar and difficult for conventional Brazilian teachers, who are accustomed to delivering lectures rather than acting as PBL tutors. To mitigate this, we have infused courses and tutor activities with both theoretical and practical aspects – including a ‘fish-bowl’ exercise, which has been an effective tactic (27).

Given the importance of the tutor's knowledge of the subjects discussed in the modules (28), another relevant topic is tutors’ preparation for the integrated content of each tutorial session. Inasmuch as our aim is to broaden the scope of discussion and the depth of student learning, we have relied on ‘content expert’ tutors to maximize confidence and minimize preparation time. Anecdotally, students have seemed approving of this change.

With regard to formative assessment, our experience has shown that many students lack the maturity to be meaningfully involved in this admittedly subjective process. Formative assessment poses challenges for tutors, as well, and requires attention and training to ensure fair and constructive formative assessment. Therefore, we leave student assessment entirely to tutors.

### Medical skills unit

Despite the aforementioned limitations of our comparatively young and inexperienced medical students, a challenge in any curricula is providing patient contact as early as possible. For this reason, the medical skills unit is responsible for placing the student in direct, supervised, contact with the medical practice during the first stage of their training – supplementing experience that can be gleaned elsewhere (29). Moreover, these placements are integrated with tutorial session content and, whenever possible, with primary care. In view of the large numbers of patients seen, we do not use simulated patients except for Objective Structured Clinical Examinations (30).

**Fig. 1:**
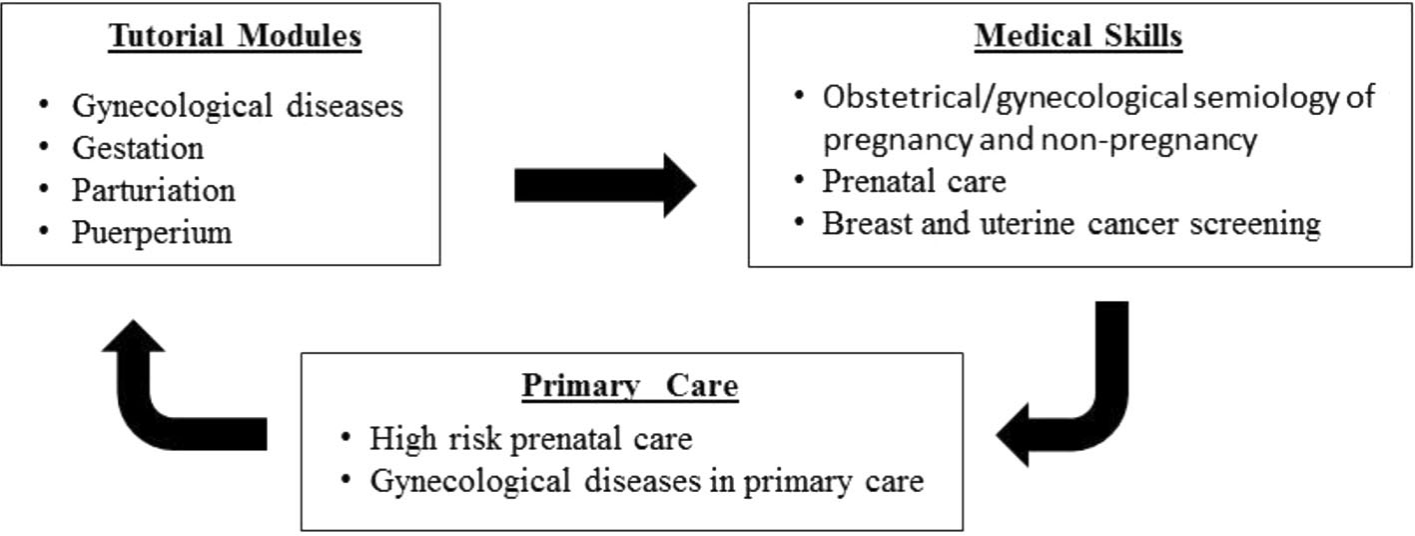
Integrating women's health content within PBL tutorial modules, medical skills, and primary care units.

Another difficulty is allowing all students to see patients with the most prevalent diseases in our region. Thus, the medical skills unit is integrated in a ‘spiral’ curriculum where topics can be revisited at strategic points. For example, during the gynecology tutorial session, students practice obstetrics and gynecological skills and see patients with gynecological diseases in the primary care setting (Fig. 1).

### Primary care unit

As in the medical skills unit, the model allows students to be integrated into communities where the FHP has been implemented. Since their first year of study, students follow health agents in home visits – exposing them to the ‘social reality’ of patients’ health problems. Along the way, students acquire the ability to deal with increasingly complex health problems; in the corresponding clerkship, they are part of the health team, serving families in health units.

Each semester, the majority of primary care-oriented activities parallel the knowledge acquired in tutorial sessions and the medical skills unit. For example, in the fourth semester, students discuss problems related to the women's health in tutorial sessions while training in the skills laboratories to perform breast, gynecologic, and obstetrical examinations. In their primary care training, students during this same semester visit pregnant and non-pregnant women to apply and reinforce these acquired knowledge and skills.

### Clerkship training

Clerkship training involves primarily bedside teaching in the inpatient wards and outpatient, ambulatory care in the specialty and primary care clinics – again, complemented by seminars, classes, clinical discussion sessions, and so on. Rotations in internal medicine, surgery, obstetrics and gynecology, pediatrics, and primary care are required. Of course, because patient types and numbers vary, and patient care must be delivered immediately, it is often neither possible nor practical to discuss specific cases in a tutorial session (i.e., discussing the case, establishing the learning objectives, engaging in self-study, and final reporting).

## Conclusion

For PBL approaches to be optimally effective, they must be adapted to the sociocultural, economic, and educational context of a particular region. This challenge is further complicated if restrictions exist its full and complete implementation – or by the planned integration of content with other curricular units or modules. However, by recognizing these potential forces, and making some strategic adaptations to our curriculum, we believe PBL-based instructional methods are a worthwhile approach to teaching Brazilian medical students and could likely be implemented in other, equally diverse training settings.
